# Letter from Dr. J. Q. Burton

**Published:** 1908-03

**Authors:** J. Q. Burton

**Affiliations:** Farwell, Texas


For the Texas Medical Journal.

### Letter From Dr. J. Q. Burton.

HIS TREATMENT FOR TYPHOID FEVER.

FARWELL, TEXAS, January 27, 1908.

*Editor Texas Medical Journal:*

After the promise to report my treatment of typhoid, I shrink from the task for the reason that the idea is so impressed on the medical mind that the disease is self-limited; that such a report would receive scant consideration, yet I will make the venture, though I have to take issue with such a distinguished man as Dr. Osler. To anticipate certain lines of criticism, I want to beg the profession not to doubt my diagnosis; rather, my truthfulness, for I claim to know what typhoid fever is, having practiced medicine for twenty-three years, and, as others, have traveled the long and thorny road of thirty, forty, and sometimes sixty days of that justly dreaded disease.

Fifteen years ago I was partially successful in the treatment of typhoid fever, reducing the time, in a great many cases, to fifteen days' duration. Then I relied mainly on large doses of subnitrate of bismuth, but, not knowing a purgative that I could always rely on, would sometimes fail, though my death rate was very low. In 1905 I added calomel to my former treatment, with uniform success, reducing the time to an average of ten days of fever.

My method, when I can positively make a diagnosis of typhoid, or in suspected cases (I do not wait for development, for valuable time may be lost), I commence with from one to three grains of calomel, divided into six parts, given half hour apart, usually in the evening, followed by castor oil and spirits of turpentine six hours later, if necessary, and this is repeated every night or every other night, according to condition of tongue and bowels, and all the way through the course of the disease. Usually, on the second visit, I make the following prescription, varying it according to age and condition of patient:

℞ Bismuth subnitrate ........................℥ss

Lac pepsin ................................ʒiss

Bicarb. soda ................................ʒi

M. Divide into charts No. 16. S. One every three hours.

I keep this prescription up as long as there is fever, and afterward further apart, till patient is up.

For the diet I rely mainly on buttermilk, and at times let them fast when nothing is relished. When I do feed, I give food regularly, every three hours in day, once at night. Peptonoids do well for a change in some cases. Do not give animal food of any kind till convalescence is well established, and no solid food for a week or ten days after fever is gone. That the fever is gone is not proof that ulcers are entirely healed. The old theory that the typhoid bacillus would keep on multiplying in the blood till the fever ran its course, regardless of the source of supply, can but be erroneous. Heal the ulcers, thereby stopping the source of the bacteria and your patient gets well.

I meet symptoms and indication as they arise, but the foregoing plan is my main reliance, and usually does the work. This report and my confidence based on same is made on twelve cases treated in the year 1905, four cases in 1906 and eleven cases in 1907. There was two deaths, one from heart failure after convalescence was established; one with symptoms of perforation, and hemorrhage of bowels, evidently a tuberculous patient. While I would like to have a larger number to base my claim on, yet these are sufficient to give me a great deal of confidence in the treatment. The shortest number of days any of these cases ran was eight and the longest was fifteen, from the time I took charge of them; had complications in a few cases. One had symptoms of cerebro-spinal irritation, lasting three weeks; no fever. Usually where fever was gone they gained quite rapidly in strength and flesh.

This report is not intended for those who think all wisdom, knowledge and discoveries must come from some noted and distinguished member of the profession. Though I would not detract and try to discredit such distinguished members, yet I claim that the humblest may make discoveries just as important to mankind as the most distinguished, and for that reason I make bold to make this report. J. Q. Burton.

